# Disparate Tuberculosis Disease Development in Macaque Species Is Associated With Innate Immunity

**DOI:** 10.3389/fimmu.2019.02479

**Published:** 2019-11-01

**Authors:** Karin Dijkman, Richard A. W. Vervenne, Claudia C. Sombroek, Charelle Boot, Sam O. Hofman, Krista E. van Meijgaarden, Tom H. M. Ottenhoff, Clemens H. M. Kocken, Krista G. Haanstra, Michel P. M. Vierboom, Frank A. W. Verreck

**Affiliations:** ^1^TB Research Group, Department of Parasitology, Biomedical Primate Research Centre, Rijswijk, Netherlands; ^2^Department of Infectious Diseases, Leiden University Medical Centre, Leiden, Netherlands

**Keywords:** tuberculosis, innate immunity, non-human primates, pulmonary immunology, experimental models of disease

## Abstract

While tuberculosis continues to afflict mankind, the immunological mechanisms underlying TB disease development are still incompletely understood. Advanced preclinical models for TB research include both rhesus and cynomolgus macaques (*Macaca mulatta* and *Macaca fascicularis*, respectively), with rhesus typically being more susceptible to acute progressive TB disease than cynomolgus macaques. To determine which immune mechanisms are responsible for this dissimilar disease development, we profiled a broad range of innate and adaptive responses, both local and peripheral, following experimental pulmonary *Mycobacterium tuberculosis* (*Mtb*) infection of both species. While T-cell and antibody responses appeared indistinguishable, we identified anti-inflammatory skewing of peripheral monocytes in rhesus and a more prominent local pro-inflammatory cytokine release profile in cynomolgus macaques associated with divergent TB disease outcome. Importantly, these differences were detectable both before and early after infection. This work shows that inflammatory and innate immune status prior to and at early stages after infection, critically affects outcome of TB infection.

## Introduction

Tuberculosis (TB), primarily caused by infection with *Mycobacterium tuberculosis* (*Mtb*) or one of the related *Mtb*-complex species, remains the leading cause of death from a single infectious agent. Annually, over 1.5 million individuals die of tuberculosis and an estimated 10 million people develop TB disease (1). Although it is assumed that some individuals are able to resist *Mtb* infection upfront, typically, successful infection can lead to acute progressive disease. The majority of infected individuals however, will control the invading *Mycobacterium* to an extent where there is no overt disease manifestation. Although it is appreciated that within an asymptomatic individual *Mtb* infection is dynamic rather than static, and that asymptomatic infection could be further differentiated into distinctive states (2–4), the majority of asymptomatically infected individuals establish a condition that can be referred to as latent TB infection (LTBI). By estimation, approximately a quarter of the world's population carries a latent *Mtb* infection (5).

Disease manifestation after *Mtb* infection is diverse, as are the dynamics of the underlying host-pathogen interactions. There is a growing range of cellular and molecular host defense and inflammatory signaling associated with anti-mycobacterial immunity and TB pathogenesis, which, therefore, is likely to affect the outcome of *Mtb* infection (3, 6–8). Despite the increasing knowledge base, we are still unable to accurately diagnose and predict who is at risk of developing TB disease and who will be able to control infection. Classically, T-lymphocyte derived IFNγ is recognized as an essential component of an effective anti-mycobacterial response and is harnessed in TB diagnostics in *Mtb-*specific IFNγ Release Assays (IGRA). Likewise, TNFα and its receptor mediated signaling are known as a critical component of effective anti-*Mtb* immunity, while recently we and others have found evidence of a possible role of IL17A in protection from TB infection and disease (9–11). However, it is becoming increasingly clear that (peripheral) adaptive T-cell immune analysis on its own is likely to be insufficient to provide sufficiently accurate correlates of protective immunity against TB (3, 6–8). Rather, a comprehensive temporal and spatial analysis of innate in addition to adaptive host response characteristics might allow identification of factors that differentiate between those at risk of active disease vs. individuals that will develop TB disease tolerance (12).

While animal models play an important role in the preclinical research and development process of new TB vaccines and therapies, they also provide great opportunity for studying immune correlates and disease mechanisms. Macaque (*Macaca* spp.) models of TB in particular recapitulate many key aspects of TB disease in humans (13–15). Cynomolgus macaques (*M. fascicularis*) and rhesus macaques (*M. mulatta*) are both used to study *Mtb* infection, but, while phylogenetically closely related, they differ significantly in their response to mycobacterial infection. In an earlier report it was shown that, in a high-dose *Mtb* challenge experiment, the efficacy of Bacillus Calmette-Guerin (BCG) vaccination differed between the two species, with vaccination conferring better protection to cynomolgus macaques (16). Subsequently, the reduced susceptibility to the development of TB-associated pathology after experimental *Mtb* infection of cynomolgus macaques compared to rhesus macaques was further established (17, 18). Furthermore, LTBI, which in these animals is characterized by sustained absence of clinical disease parameters and bacteria in bronchoalveolar or gastric lavage, occurs in approximately half of cynomolgus macaques upon low dose infection with 25–50 colony forming units (CFU) of *Mtb* (19). Development of LTBI in rhesus macaques, however, has not been reported yet. A notable exception to these findings is the Mauritian cynomolgus macaque, a genetically distinct population of cynomolgus macaques with limited major histocompatibility complex (MHC) diversity, which appear to be equally susceptible to TB disease as rhesus macaques (18, 20).

The difference in TB disease susceptibility between rhesus and cynomolgus macaques has been well-described and, yet, the host response mechanisms that determine this differential outcome of *Mtb* infection are poorly understood. To the best of our knowledge, only two studies compared rhesus and cynomolgus macaques head-to-head for their susceptibility to disease after *Mtb* challenge (17, 18). In one of these studies a comparative immune analysis between the two species was reported, which however, was limited to a reduced IFNγ response signal from peripheral blood mononuclear cells (PBMC) in association with reduced TB disease severity. Furthermore, it remains unresolved if there is a difference in susceptibility to *Mtb* infection as well.

In the study reported here, we sought to identify the minimal infectious dose (21) for either of the species, while simultaneously profiling both peripheral as well as local adaptive and innate immune responses, to identify responses associated with and possibly predicting differential susceptibility to TB disease. We show comparable time and dose response dynamics to infectious *Mtb* challenge and corroborate the differential disease susceptibility between the two species. Most importantly, our immune analysis shows that rhesus macaques display anti-inflammatory monocyte skewing in the periphery, while cynomolgus macaques show a higher production of inflammatory cytokines locally, prior to and early after *Mtb* exposure.

This suggests that early orchestration of pro- and anti-inflammatory innate responses are underlying the distinctive TB disease development between cynomolgus and rhesus macaques after low dose *Mtb* infection, providing important insights in immune correlates of susceptibility to TB disease as well as mechanisms of early control of *Mtb* infection.

## Results

### Infection Take After Repeated Exposure to Increasing Doses of *Mtb*

**Figure 1:**
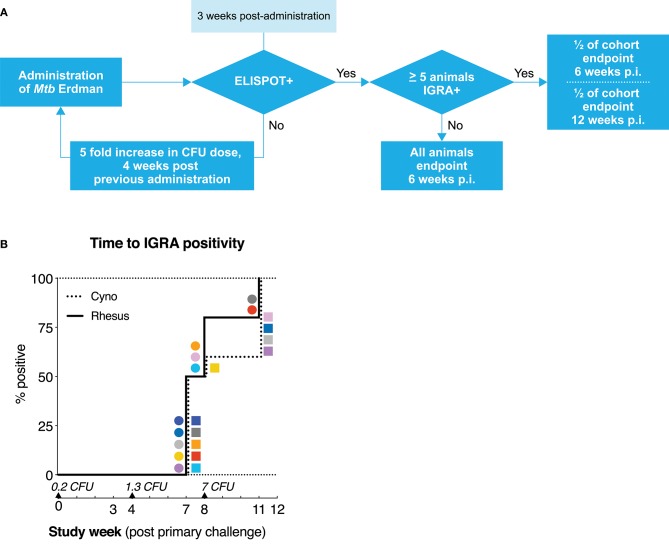
*Mtb* dose escalation challenge in cynomolgus and rhesus macaques. **(A)** Schematic representation of the minimal *Mtb* dose-finding strategy applied in this study. Rhesus and cynomolgus macaques, *n* = 10 each, were endobronchially challenged with an initial dose of 0.2 CFU of *Mtb* strain Erdman, after which infection take was determined by means of an *Mtb* specific IFNγ ELISPOT. Non-infected (read: IGRA–) animals were challenged with an increasing dose of *Mtb* until ELISPOT-positivity was obtained. ELISPOT-positive animals were randomly assigned and sacrificed either at 6 (*n* = 6 per species) or 12 weeks (*n* = 4 per species) after infectious *Mtb* exposure. **(B)** Kaplan-Meier curve depicting the rate of IFNγ ELISPOT positivity/IGRA conversion. *Mtb* challenge events are indicated by triangles along the x-axis, accompanied by the calculated (extrapolated) challenge dose, verified by quality control plating. Squares represent individual cynomolgus macaques, circles represent individual rhesus macaques. Color coding per individual, as defined in Table 1, is consistently applied throughout.

Cynomolgus and rhesus macaques present with differential susceptibility to pathology after *Mtb* infection. To investigate if the two species also differ in susceptibility to infection, we designed a dose-escalation study. Animals (*n* = 10 per species) were exposed to increasing doses of *Mtb* strain Erdman K01 by endobronchial installation and monitored for success of infectious challenge at regular intervals. By study design, challenge events were planned at 4 weeks intervals and whether infectious challenge was successful was monitored 3 weeks after each *Mtb* instillation [based on previous *Mtb* challenge studies (22)]. As a surrogate for successful infection, hereafter referred to as infection-take, we used a negative to positive conversion in an IGRA, in this case a NHP-specific IFNγ ELISPOT lab test to be specific. The threshold for IGRA conversion was set at the average pre-infection response to *Mtb* Purified Protein Derivate (PPD) or ESAT6-CFP10 fusion-protein plus three times the standard deviation (in this case, 50 spots per million cells). When no infection-take was observed 3 weeks after challenge, by study design, animals were exposed to a 5-fold increased dose of *Mtb* the following week (Figure 1A). At the same time, another ELISPOT was performed at that point, again, to monitor (delayed) infection-take. Exposure to *Mtb* was repeated until all animals had an established infection. When ≥5 animals IGRA converted after a given dose of *Mtb*, that cohort was divided in two and sacrificed either 6 or 12 weeks after infection, to compare “early” vs. “late” stage pathology and immunology.

The initial challenge dose was set at less than a single CFU of *Mtb* per dose (on average) to ensure we would capture a condition at which infection-take was a matter of chance. After administration of a calculated average of 0.2 CFU, none of the animals showed conversion to IGRA positivity (Figure 1B). After the subsequent, increased challenge with a calculated average of 1.3 CFU of *Mtb*, IGRA conversion occurred in half of the animals of both species at 3 weeks post-challenge. One week later, an additional 3 rhesus macaques and 1 cynomolgus macaque converted to IGRA+. In accordance with the study design, these latter animals had received the next dose of 7 CFU of *Mtb* at that time point. Thus, these four animals are formally considered to be “superinfected.” Additionally, one IGRA+ cynomolgus macaque reverted to become IGRA– (C9, dark gray). As this transient IGRA+ animal had met the pre-defined criteria for infection, it was treated as such for the remainder of the study and, therefore, did not receive additional doses of *Mtb*. All remaining IGRA– animals (2 rhesus and 4 cynomolgus) became IGRA+ after the third exposure event to 7 CFU of *Mtb*. An overview of the infectious dose and time of IGRA conversion per animal is given in Table 1. With the exception of the one reverting cynomolgus macaque (C9, dark gray) all animals remained IGRA+ from their conversion time point onward. Statistical analysis of the rate of IGRA conversion revealed no significant difference in conversion dynamics between cynomolgus and rhesus macaques. Although sample size (*n* = 10 per species) was limited, the data suggest that both species are similarly susceptible to *Mtb* infection, as measured by IGRA conversion.

**Table 1:** Infection characteristics after dose-escalation *Mtb* administration for each individual.

	**Cynomolgus**									
Animal ID	C1	C2	C3	C4	C5	C6	C7	C8	C9	C10
Symbol	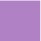	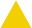	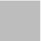	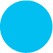	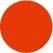	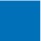	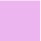	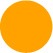	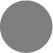	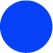
Infectious dose (CFU)	7	1	7	1	1	7	7	1	1	1
IGRA+ at week	11	8	11	7	7	11	11	7	7	7
Superinfected (y/n)	n	y	n	n	n	n	n	n	n	n
Sacrificed at week	12	6	12	6	12	6	6	6	12	6
	**Rhesus**									
Animal ID	R1	R2	R3	R4	R5	R6	R7	R8	R9	R10
Symbol	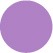	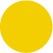	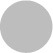	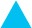	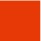	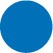	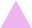	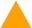	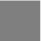	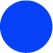
Infectious dose (CFU)	1	1	1	1	7	1	1	1	7	1
IGRA+ at week	7	7	7	8	11	7	8	8	11	7
Superinfected (yes/no)	n	n	n	y	n	n	y	y	n	n
Sacrificed at week	6	6	6	6	12	12	6	6	12	12

*Overview of infectious challenge dose (in CFU) that led to IGRA conversion, week of IGRA conversion, occurrence of superinfection and time to sacrifice for each individual animal*.

### Tuberculosis Disease and Bacterial Load After Low Dose *Mtb* Challenge

By study design, endpoint of post-infection follow-up was set at 6 weeks (*n* = 6/species) or 12 weeks (*n* = 4/species) after the challenge that resulted in IGRA conversion. This approach enabled host response profiling at an “early” and “later” time point (see Table 1 for time of sacrifice for each animal). For data analysis, the animals “superinfected” with 7 CFU were kept in the same arm of study design and analysis as the animals that converted after 7 CFU. At endpoint, macroscopic TB pathology in lungs, lung draining lymph nodes and extra-thoracic organs was scored by using a predefined, arbitrary scoring algorithm based on lesion size, appearance, and frequency.

**Figure 2:**
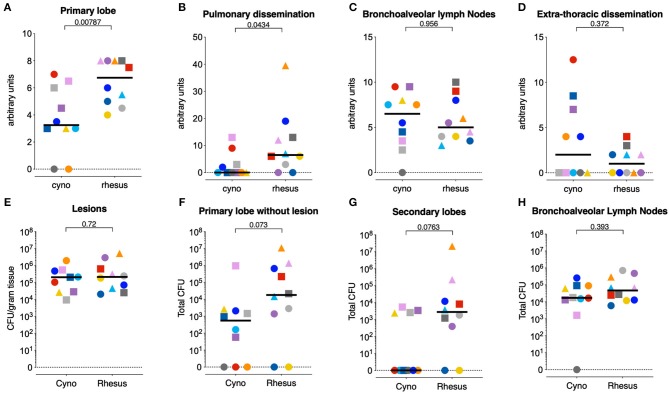
Cynomolgus macaques exhibit less pulmonary tuberculosis disease after low dose *Mtb* infection. **(A–D)** Post-mortem scores of gross *Mtb* pathology in **(A)** the primary targeted lung lobe, targeted when challenging with *Mtb*, **(B)** all other lung lobes, as pulmonary dissemination, **(C)** the lung-draining lymph nodes, and **(D)** the extra-thoracic organs as extra-thoracic dissemination. **(E–H)** Quantification of bacterial load in homogenates **(E)** of individually collected and pooled lesions from the primary lobe, **(F)** of the remainder of the primary lung lobe (after collection of individual lesions), **(G)** of pooled secondary lung lobes, and **(H)** of lung draining lymph nodes. Circles represent animals that IGRA converted 3 weeks after 1.3 CFU challenge, squares represent animals that IGRA converted 3 weeks after 7 CFU challenge, and triangles represent animals that IGRA converted 4 weeks after 1.3 CFU challenge and received another 7 CFU of *Mtb* 1 week later. *N* = 10 per group, except for figure **(E)**, where *n* = 9 cynomolgus macaques, as one animal did not yield any macroscopic lesions. Horizontal lines indicate group medians. Significance of differences between species was determined by two sided Mann-Whitney testing. Color coding per individual, as defined in Table 1, is consistently applied throughout.

Quantification revealed a significantly higher level of pathology in the lungs of rhesus as compared to cynomolgus monkeys, both in the primary targeted lung lobe, the site of endobronchial instillation, as well as in the secondary lung lobes as a measure of intrapulmonary dissemination (Figures 2A,B). This species difference in lung pathology was already apparent 6 weeks after infection (*p* = 0.013 for primary lobe pathology, Supplemental Figure 1a). While median lung pathology scores appeared higher 12 weeks post-infection compared to 6 weeks post-infection in both species, group sizes were too small to establish statistical significance of this increase. Interestingly, pathology of lung draining lymph nodes did not differ between the two species (Figure 2C). Extra-thoracic pathology was observed in an equal number of rhesus and cynomolgus macaques (5 vs. 5, Figure 2D), and primarily affected liver and spleen. When present, cynomolgus monkeys generally exhibited more severe extra-thoracic pathology. The different infectious doses (1.3 vs. 7 CFU), regardless of species, were not found to be associated with pathology levels.

*Mtb* tissue burden was quantified by plating homogenates of specific lesions sampled from the primary, targeted lung lobe, the remainder of the primary lobe, the pooled secondary lobes, and the lung draining lymph nodes. When assessing *Mtb* burden in granulomas isolated from the targeted lung lobe, we did not detect a difference in CFU between the two species (Figure 2E), as described previously (18). However, bacterial burden in the remainder of the primary lobe as well as the secondary lung lobes trended to be lower in the cynomolgus macaques compared to the rhesus macaques, in accordance with the extent of observed lung pathology (Figures 2F,G). CFU counts from lung draining lymph nodes did not differ between groups (Figure 2H). The cynomolgus macaque that was transiently IGRA+ (C9, dark gray) had no detectable pathology nor CFU in the organs analyzed. Whether this animal was not infected or cleared the infection at a very early stage remains unclear. Consequently, this animal was excluded from the downstream immune analyses.

In addition to pathology and bacterial burden, we measured several clinical parameters associated with progressive TB disease in NHP. No marked weight loss was observed in either group (Supplemental Figure 1b), with the exception of one rhesus macaque, which also reached a humane endpoint due to relatively severe disease development (R8, orange). Furthermore, over time, neither the mean corpuscular hemoglobin (MCH) and C-reactive protein (CRP) levels, as markers of infection-associated anemia or systemic inflammation, respectively, nor the monocyte/lymphocyte ratio (23), showed any difference between the two species (Supplemental Figures 1c–e). This lack of differences in clinical features of experimental TB disease is in accordance with the low challenge dose and the relatively short follow-up time post-infection in this experiment.

### *In vitro* Mycobacterial Growth Control After *Mtb* Infection

**Figure 3:**
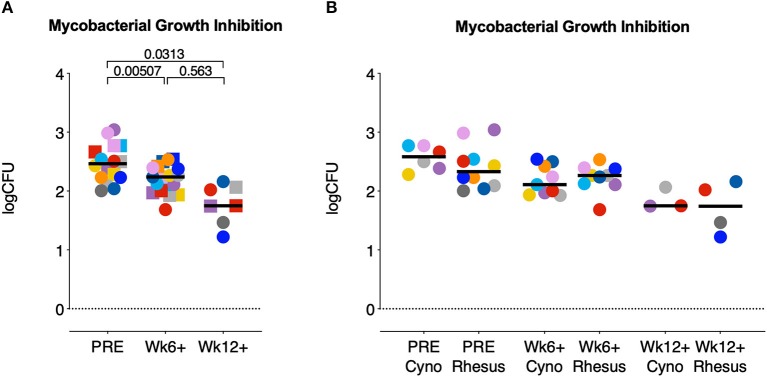
Mycobacterial growth inhibition capacity increases after *Mtb* infection but does not differ between species. A mycobacterial growth inhibition assay (MGIA) was used to determine the potential of rhesus and cynomolgus PBMC to control mycobacterial growth after infection. Data are depicted relative to individual IGRA conversion time points, for **(A)** rhesus and cynomolgus together, or **(B)** for each of the two species separately. Number of animals per time point varied due to sample availability. Horizontal lines indicate group medians. In **(A)**; squares represent cynomolgus samples, circles represent rhesus samples. Two-sided Mann-Whitney testing was used to determine significance of differences between groups. Color coding per individual, as defined in Table 1, is consistently applied throughout.

After low dose *Mtb* infection cynomolgus macaques display a reduced amount of lung pathology and a lower mycobacterial load compared to rhesus monkeys. To investigate whether control of bacterial replication could underlie this milder disease phenotype, we employed a mycobacterial growth inhibition assay (MGIA) (24). PBMCs from both species, isolated pre-infection, 6 weeks post-infection and, where applicable, 12 weeks after infection, were co-cultured for 96 h with BCG and subsequently transferred to Mycobacterial Growth Indicator Tubes (MGITs), as detailed in Tanner et al. (25), to compare the rates of mycobacterial outgrowth. As observed previously in humans with PBMCs from recently *Mtb* infected vs. uninfected humans (26), BCG outgrowth was significantly reduced after incubation with cells obtained post-*Mtb* infection in comparison to cells obtained from the same animals pre-infection (Figure 3A). However, when comparing the outgrowth between species no differences between the two groups were found (Figure 3B). Thus, the reduced pathological involvement and bacterial tissue burden observed in cynomolgus compared to rhesus macaques is not reflected in differential bacterial outgrowth control as measured by PBMC-based MGIA.

### Local and Peripheral T-cell Responses After Low Dose *Mtb* Challenge

In an effort to identify potential immune correlates associated with the difference in disease development between the two species, we profiled peripheral as well as local adaptive immune responses at various timepoints after *Mtb* infection-take (as measured by IGRA conversion). Consequently, all data are aligned to the moment of each individual's time point of infection-take.

**Figure 4:**
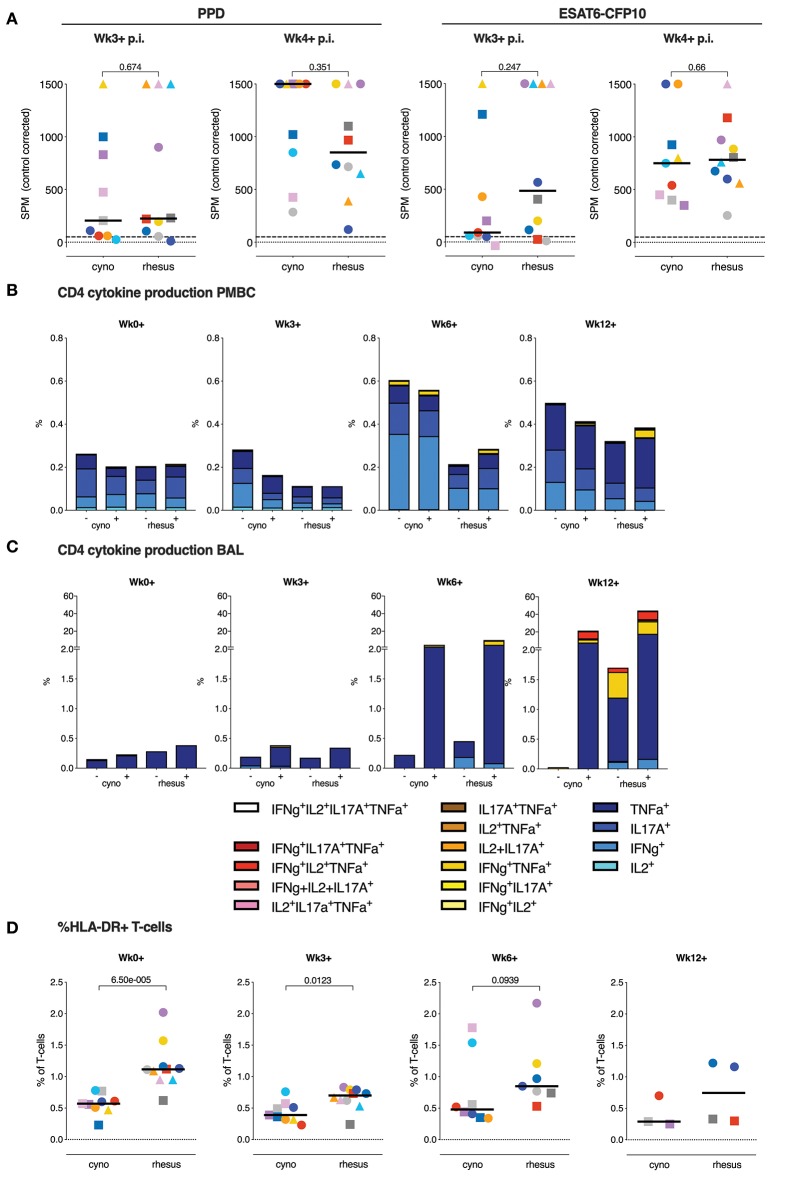
T lymphocyte cytokine responses do not associate with the differential disease outcome between species. **(A)** The IFNγ ELISPOT response to PPD and ESAT6-CFP10 recall stimulation *in vitro*, 3 or 4 weeks post-*Mtb* infection take (p.i.; median of triplicates per animal). **(B)** Median frequency of cytokine producing CD4+ T-cell subsets in PBMC, and **(C)** in BAL, both after overnight stimulation with culture medium as a control (–) or *Mtb*-derived PPD (+). **(D)** Frequencies of circulating HLA-DR+ T-cells. Data are aligned to the moment of each individual's time point of infection take. Number of animals per time point varies due to sample availability. Horizontal lines indicate group medians in **(A,D)**; dashed lines in **(A)** mark the cut-off value for positivity in the ELISPOT assay. *P*-values of possible differences between species was determined by two-sided Mann-Whitney testing. Color coding per individual, as defined in Table 1, is consistently applied throughout.

Beyond assessment of IGRA conversion, quantification of the IFNγ ELISPOT responses measured 3 and 4 weeks after infection revealed no differences in the capacity of PBMCs of rhesus and cynomolgus macaques to produce IFNγ in response to either PPD or ESAT6-CFP10 stimulation (Figure 4A).

PPD-specific production of IFNγ, TNFα, IL2, and IL17A by peripheral CD4+ T-cells was assessed by flow-cytometry. Over time, low dose *Mtb* infection appeared to induce little detectable PPD-specific, peripheral cytokine production in either species, and there were no appreciable differences in frequencies or polyfunctionality observed between the two species (Figure 4B and Supplemental Figure 2a). Transient elevation of background cytokine production was observed in cynomolgus macaques, which remains unexplained, especially since it occurs in the absence of an appreciable induction of PPD-specific responses, but may reflect some systemic increase in the activation status of CD4+ T lymphocytes in the blood.

Profiling of the local response by BAL cell analysis revealed the induction of substantial frequencies of PPD-specific cytokine+ CD4+ T-cells from 6 weeks post-infection onward, with on average strongest response signals at 12 weeks post-infection (Figure 4C and Supplemental Figure 2b). Despite marginal differences occasionally, also for the local CD4 T-cell response in the bronchoalveolar space, it appeared that neither magnitude nor phenotype seemed to be associated with the differential pathology levels between rhesus and cynomolgus macaques. Due to a technical error CD8+ T-cells in BAL samples were not stained directly. However, analysis of CD4 negative CD3+ cells, which contain the unstained CD8+ T-cells, again, revealed no significant differences in cytokine production between two species (data not shown). At later timepoints cynomolgus macaques trended toward lower T-cell responses in the lung, potentially reflecting reduced T-cell priming due to lower antigenic load.

Intriguingly, we did observe a higher frequency of circulating HLA-DR+ T-cells, a known correlate of an increased TB disease risk in man (27), in rhesus compared to cynomolgus macaques at the moment of and early after *Mtb* infection (Figure 4D).

Collectively, peripheral as well as local *Mtb-*specific Th1 and Th17 responses, within the limits of our analyses, appeared not to be implicated in the differential disease susceptibility observed between rhesus and cynomolgus macaques.

### Post-*Mtb* Infection Humoral Immune Responses in the Lung and Periphery

We subsequently investigated if the humoral arm of the adaptive immune response was associated with the difference in TB manifestation between the species, as *Mtb*-specific antibodies can potentially contribute to mycobacterial infection control in various ways (28, 29). The *Mtb*-specific antibody response was assessed both peripherally and locally by measuring *Mtb* whole cell lysate (WCL) specific IgM, IgA, and IgG in serum and BAL fluid collected over the course of the infection.

**Figure 5:**
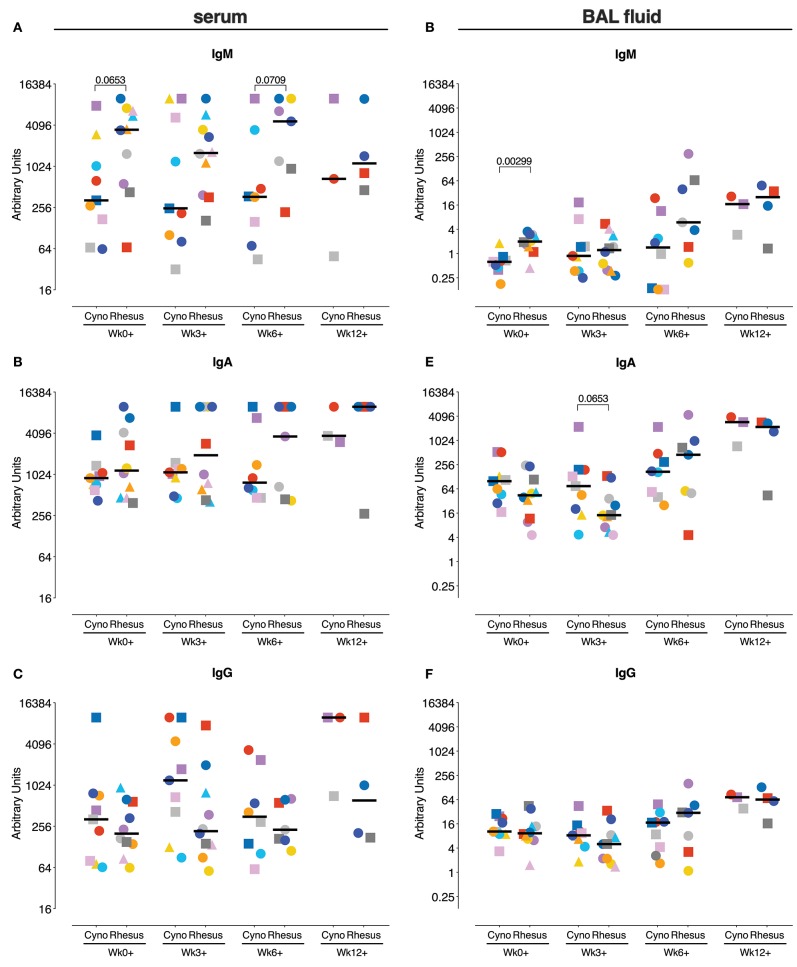
Antigen-specific antibody levels after *Mtb* infection. Measurement of *Mtb-*specific IgM **(A,D)**, IgA **(B,E)**, and IgG **(C,F)** antibody response in serum **(A–C)** and BAL fluid **(D,E)** prior to and after *Mtb* infection. Antibody levels are plotted as arbitrary units, determined by standardization against a reference sample. Data are aligned to the moment of each individual's time point of infection take. Number of animals per time point varies due to sample availability. Horizontal lines indicate group medians. Significance of group differences was determined by two-sided Mann-Whitney test. Color coding per individual, as defined in Table 1, is consistently applied throughout.

Prior to infection with *Mtb*, rhesus macaques showed a trend toward higher levels of *Mtb*-reactive serum IgM, though not IgA or IgG (Figures 5A–C). After *Mtb* infection, the serum antibody responses to *Mtb* were increased in some, but not all animals and serum IgM levels were higher in rhesus monkeys compared to cynomolgus macaques, although this difference just failed to reach statistical significance. No species-related differences were apparent in the magnitude of the *Mtb*-specific IgA and IgG response after *Mtb* infection (Figures 5B,C).

Locally, again, higher *Mtb*-specific IgM levels prior to infection were observed in rhesus macaques (Figure 5D), while no differences were observed in *Mtb*-specific IgA and IgG responses (Figures 5E,F). Like in serum, *Mtb*-specific antibody-levels in BAL seemed to increase in some but not all animals over the course of *Mtb* infection. The magnitude of the local antibody responses during *Mtb* infection does not significantly differ between the species at any time point.

### Peripheral Monocyte Phenotype After *Mtb* Infection

As our adaptive T cell and humoral response analysis revealed no overt differences between rhesus vs. cynomolgus macaques, we next investigated if a differential innate immune response could be underlying the disparate disease susceptibility between the species. We stimulated whole blood with *Mtb* WCL and assessed cytokine production by different Antigen Presenting Cell (APC) subsets by flow cytometry. *Mtb* WCL contains the full range of Pathogen Associated Molecular Patterns (PAMPs) of *Mtb* and therefore should activate several of the Pathogen Recognition Receptors (PRRs) of the innate immune system. We profiled production of three key cytokines, TNFα, IL12, and IL8, in monocytes and DC subsets before and at 3, 6, and 12 weeks after infection.

**Figure 6:**
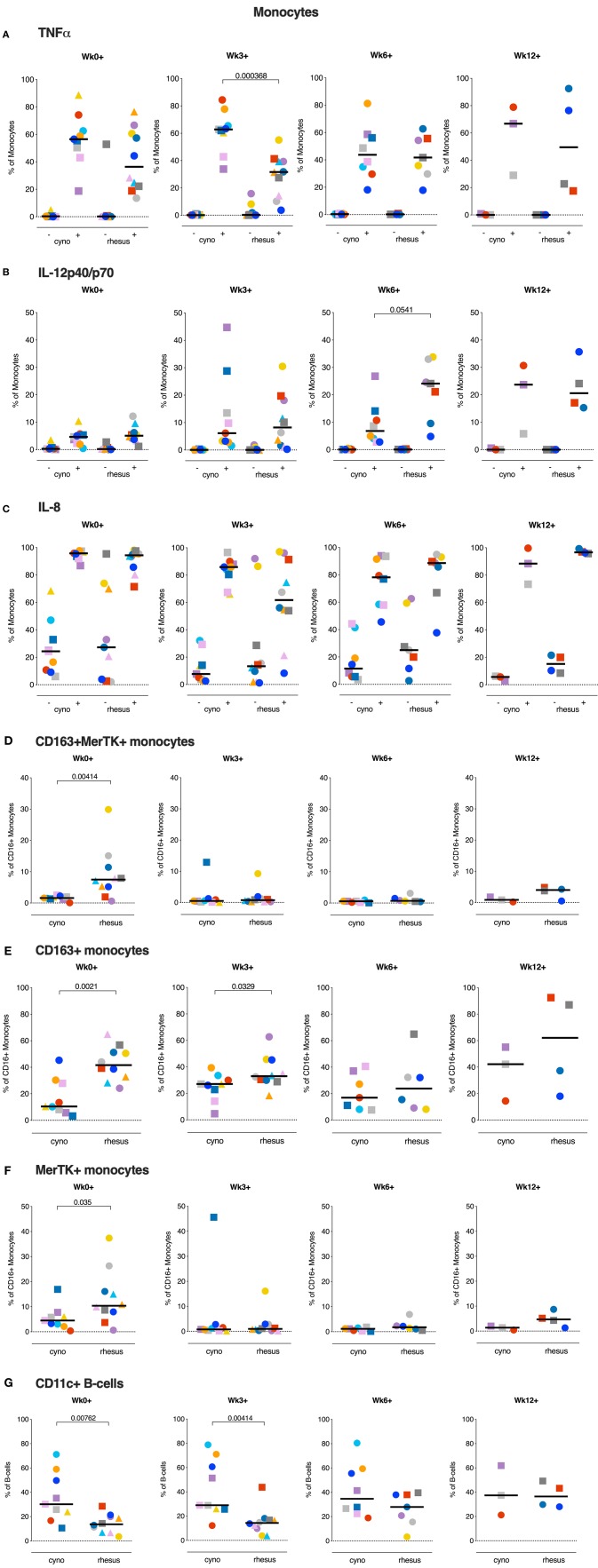
Monocytes of cynomolgus and rhesus macaques show different functional phenotypes before and after *Mtb* infection. **(A–C)** Percentage TNFa, IL12p40/p70, and IL8 positive monocytes of total CD14+ monocytes after overnight stimulation of peripheral whole blood with culture medium (–) or *Mtb* Whole Cell Lysate (+). **(D–F)** *ex vivo* expression of CD163 and MerTK on CD16+ monocytes from peripheral whole blood. **(G)** Percentage of CD11c+ B-cells in peripheral whole blood. Data are aligned to the moment of each individual's time point of infection take. Number of animals per time point varies due to sample availability. For **(D–F)**; samples that contained <100 events in the CD16+ monocyte gate were excluded from analysis. Horizontal lines indicate group medians. Significance of group differences was determined by two-sided Mann-Whitney test. Color coding per individual, as defined in Table 1, is consistently applied throughout.

In the DC subsets, differentiated by CD11c, CD1c, and CD16 expression, we could not identify any differences in cytokine positivity (data not shown). However, in the CD14+ monocyte population we found dissimilar frequencies of cytokine producing monocytes between rhesus and cynomolgus macaques. Early after infection (Wk3+), TNFα producing monocytes were found to be more frequent in peripheral blood of cynomolgus macaques, and the frequency of TNFα positive monocytes at this time point correlated negatively with the amount of lung pathology (Spearman's rho = −0.5507, *p* = 0.0145). This difference was no longer observed at 6 weeks after *Mtb* infection (Figure 6A). At this timepoint the frequency of IL12p40/p70 positive monocytes however, was higher in rhesus macaques compared to cynomolgus macaques (Figure 6B). The frequency of IL12 producing monocytes in response to *Mtb* WCL stimulation increased over the course of the infection. Overall, frequencies of IL8 positive monocytes were high after stimulation with *Mtb* WCL, but never differed between species at any week post-infection (Figure 6C).

Monocytes display functional plasticity and are able to act in a pro- as well as an anti-inflammatory manner depending on environmental stimuli. Previously, anti-inflammatory CD16+ monocytes expressing CD163 and Mer Tyrosine Kinase (MerTK) were found to be expanded after *Mtb* exposure and associated with disease severity in man as well as macaques (30). Prompted by the differences in monocyte cytokine production we profiled the expression of these markers on circulating CD16+ monocytes of both species. When assessing the *ex vivo* frequencies of CD163+ and MerTK+ double positive CD16+ monocytes we found that already at baseline rhesus macaques had significantly higher frequencies of MerTK+CD163+CD16+ monocytes, which were however markedly decreased from 3 weeks post-infection onward (Figure 6D). Analysis of CD163+ and MerTK+ CD16+ monocytes separately (Figures 6E,F), showed that primarily the frequencies of MerTK+ CD16+ monocytes were decreased. Frequencies of circulating CD163+CD16+ monocytes, regardless of MerTK expression, remained higher in rhesus macaques up until week 3 post-infection. Statistical analysis revealed a positive correlation between the frequency of CD163+ monocytes at baseline and the amount of lung pathology (Spearman's rho = 0.4548, *p* = 0.0504).

Taken, together, these data show a more potent pro-inflammatory APC signature in cynomolgus macaques before and/or early after *Mtb* infection compared to rhesus macaques.

In addition to their capacity to produce antibodies, B-cells can also function as APCs. Atypical, CD11c+ expressing B-cells in particular have been described as potent APCs and were also found to be upregulated after TB infection (31, 32). We therefore compared the frequencies of circulating CD11c+ B-cells between two species before and after *Mtb* infection. Interestingly, a significantly larger portion of the B-cells of cynomolgus macaques displayed this atypical phenotype, as characterized by CD11c expression (Figure 6G). This difference was maintained up to 3 weeks post-infection, after which the frequency of atypical B-cells in rhesus macaques starts to increase, reminiscent of what is seen in humans with TB disease. Therefore, while the post-*Mtb* antibody response did not notably differ between the species (Figure 4), B-cells in their capacity as APCs might influence disease outcome in NHPs.

### Local Innate Cytokine Production in Response to *Mtb*

In the face of aerogenic *Mtb* infection, the first immune cell encountered by the bacteria is likely to be the alveolar macrophage, the most predominant cell-type lining the pulmonary mucosa (33). These local macrophages are likely to play a critical role in determining the fate of invading *Mtb*. However, due to high auto-fluorescence flow cytometric analysis of alveolar macrophages is challenging. Therefore, to assess possible species differences in the local innate immune response, we stimulated the total of unfractionated cells obtained from BAL with *Mtb* WCL and assessed cytokine production before and after *Mtb* infection by means of a multiplex Luminex assay.

**Figure 7:**
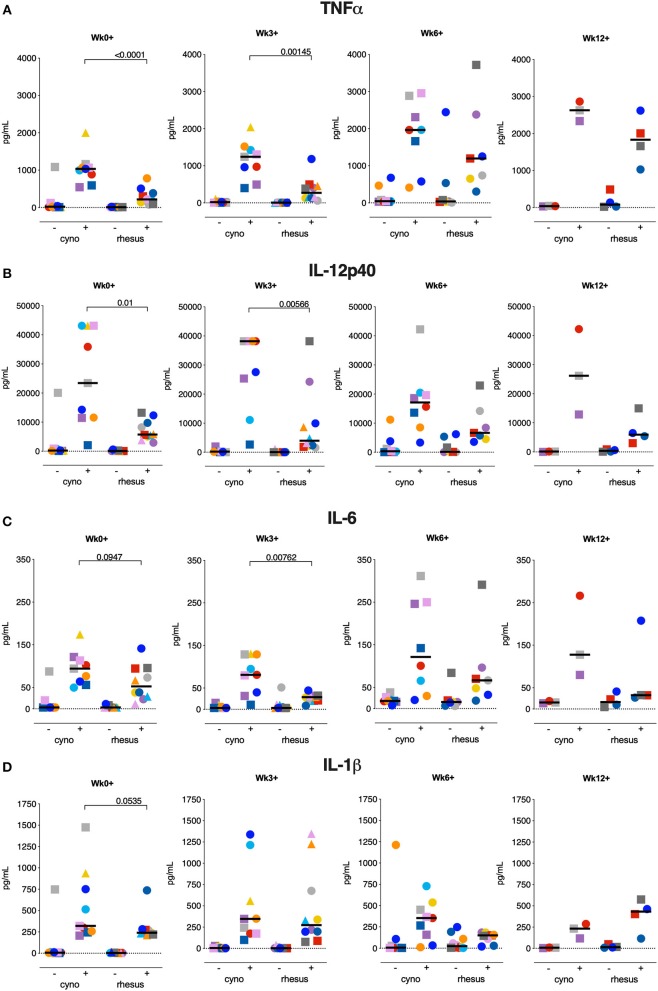
Local innate cytokine production at the time of or early after *Mtb* infection is more prominent in cynomolgus macaques. Production of **(A)** TNFα, **(B)** IL6, **(C)** IL12p40, and **(D)** IL1β by BAL cells after overnight stimulation with culture medium (–) or *Mtb* whole cell lysate (+). Data are aligned to the moment of each individual's time point of infection take. Number of animals per time point varies due to sample availability. Horizontal lines indicate group medians. Significance of group differences was determined by two-sided Mann-Whitney test. Color coding per individual, as defined in Table 1, is consistently applied throughout.

Interestingly, in response to *Mtb* WCL stimulation, TNFα, IL6, IL12p40, and IL1β were produced to a significantly higher extent by BAL cells of cynomolgus macaques already prior to infection (Figures 7A–D), concordant to the pro-inflammatory profile of circulating monocytes in cynomolgus macaques. Early after infection, TNFα, IL6 and IL12p40, but not IL1β, remained present at significantly higher levels in BAL cell supernatants from cynomolgus macaques. At later time-points differences in cytokine production between the two species could no longer be observed.

Thus, the local inflammatory response observed at the moment of and shortly after *Mtb* infection in cynomolgus macaques seems to provide a milieu that supports constraint of *Mtb*-associated pathology.

## Discussion

Rhesus and cynomolgus macaques are known to differ in their susceptibility to TB disease after *Mtb* challenge, with rhesus monkeys generally exhibiting more severe TB pathology compared to cynomolgus macaques (17, 18). Here, we demonstrate that the two species do not differ in their susceptibility to *Mtb* infection, as measured by IGRA conversion, and that administration of a single CFU of *Mtb* Erdman is sufficient to establish infection in both rhesus and cynomolgus macaques. Exposure to 1 CFU resulted in infection of 50–80% of macaques as defined by the induction of an antigen-specific IFNγ secretion response (IGRA conversion). Based on this finding we have recently defined for the first time in NHP (9) a repeated limiting dose challenge model for the readout of preclinical vaccine efficacy not only by signals of prevention of disease but also by signals of prevention of infection. As the current study demonstrates that both species are equally susceptible to *Mtb* infection and corroborates the distinct difference in TB disease susceptibility between the two species (17, 18), it also reveals differential immune response profiles that associate with and potentially underlie the differences in TB pathogenesis. Cynomolgus macaques exhibited a stronger local pro-inflammatory response than rhesus monkeys, while rhesus macaques displayed a more anti-inflammatory peripheral monocyte phenotype. Of note, the majority of the differential immune responses were observed already prior to or early after *Mtb* infection.

As both the pathogenesis and immune responses after *Mtb* infection closely resemble the spectrum of TB manifestation in man, we can consider both rhesus and cynomolgus macaques as highly relevant model species for TB research. When assessing prophylactic or therapeutic interventions rhesus macaques may be the more stringent and practical model, considering the rapid disease progression in this species. Cynomolgus macaques on the other hand would be more appropriate when investigating chronic TB manifestations or when evaluating therapies targeted to latently TB infected individuals.

In search of immune-mechanisms associated with the differential disease susceptibility between the two macaque species, we extensively profiled the adaptive immune responses known to be critically involved in anti-*Mtb* immunity. We extended our analysis beyond the conventional IFNγ (34) response by measuring TNFα, IL2, and IL17a producing T-cells as well, both in the periphery as well as at the site of the infection at various time points after infection. Peripherally, we observed little induction of *Mtb*-specific cytokine responses in either species. Profiling of the T-cell response in BAL after *Mtb* infection revealed the induction of robust T-cell responses locally, as observed previously (9). However, these responses did not discriminate between rhesus and cynomolgus macaques and therefore did not correlate with their differential disease susceptibility. Despite a non-significant difference in pre-infection IgM, but not IgA or IgG, levels, we also did not find the magnitude of the peripheral or local antibody-response to *Mtb* to differ between the species.

Within the limits of our analyses this study fails to show any significant difference in adaptive immune response parameters, but rather points to more upstream and very early immune cascades that seemingly impact on the disparate outcome of infection in cynomolgus vs. rhesus macaques.

When assessing innate immune responses in both species we found responses in the monocyte compartment in particular to be associated with differential disease outcome after low dose *Mtb* infection. Monocytes and macrophages, alveolar macrophages in particular, are considered to be the typical target cell for *Mtb* infection, where the bacterium, by interfering with macrophage effector function, can proliferate and persist (35). Perturbation of the monocyte compartment in TB patients has been associated with expansion of a suppressive, CD16+, non-classical monocyte population exhibiting an anti-inflammatory phenotype, characterized by the expression of CD163 and MerTK (30, 36). These CD16+CD163+MerTK+ monocytes were found to be more permissive to mycobacterial growth. In rhesus macaques, we found greater levels of circulating CD163+CD16+ monocytes prior to and early after *Mtb* infection. Furthermore, the frequency of circulating monocytes expressing MerTK, a negative regulator of T-cell responses (37), was found to be higher in rhesus macaques prior to *Mtb* infection. Although no difference in T-lymphocyte effector populations could be found in the present study, the increased frequency of MerTK+ monocytes in rhesus macaques could affect a T-cell population beyond the (detection-)limits of the analyses performed and samples profiled in this study. Intriguingly, in addition to the distinct monocyte response between the species, we also found a population of atypical, CD11c expressing B-cells, described as potent APCs, to be more frequent in cynomolgus macaques. While B-cell depletion in cynomolgus macaques did not have an unequivocal effect on *Mtb* infection and disease, granulomas of B-cell depleted monkeys tended to contain more bacilli (38). The finding that, in individuals with active TB, B-cells display a dysfunctional phenotype and significantly impact *Mtb*-specific T-cell responses further points to a role for B-cells in the protective immunity against TB (32).

Of note, TNFα production by innate immune cells, both peripherally and locally, was found to be higher in the more disease-resistant cynomolgus macaques early after *Mtb* exposure. TNFα has been identified as a crucial component in the anti-mycobacterial immune response, playing a role in, amongst others, granuloma formation and APC activation (39). The importance of TNFα is maybe best emphasized by the observation that patients receiving anti-TNFα treatment are at an increased risk of disseminating reactivation TB (40). Interestingly, in mice myeloid derived TNFα specifically was required for early control of *Mtb* replication, while T-cell derived TNFα was necessary for control of chronic TB infection (41).

In addition to TNFα, proinflammatory IL1β, IL6, and IL12p40 were found to be secreted to a greater extent by (non-purified) total BAL cells from cynomolgus macaques, all cytokines which have been implied previously to have a protective effect after *Mtb* infection. Knock-out studies in mice have demonstrated that IL1β is critical in host resistance to *Mtb* [reviewed in (42)] while in a Malawi cohort of pulmonary TB patients impaired IL1β (and TNFα) production in response to heat killed *Mtb* was associated with poor infection outcome (43). The IL12 cytokine family has similarly been described as a critical mediator of anti-*Mtb* resistance [reviewed in (44, 45)]. Interestingly, IL12p40 may contribute to protection not only by initiation and maintenance of a Th1 and Th17 response but also potentially by mediating activation of dendritic cells (46). The role of IL6 in anti-tuberculosis immunity is less well-defined, but a number of studies suggest a role for IL6 in *Mtb* control (42, 47, 48).

In our analysis, standard correlation analysis identified the frequency of CD163+ monocytes at baseline and the frequency of TNFa+ monocytes at 3 weeks post-infection only as statistical correlates of TB pathology. As TB disease pathogenesis is multifactorial and complex, an unbiased, multivariate analysis might be more suited to identify a protective vs. pathogenic immune signature, however the low number of samples in this study hampers such an analysis. Furthermore, sampling at even earlier timepoints, ranging from hours to several days after *Mtb* infection, would be required to further detail the role of early innate immune responses in infection outcome. Purification of specific myeloid subsets (e.g. alveolar macrophages) would add to unraveling such cascades. Taken together, the pro-inflammatory immune environment present at the mucosal surfaces at the moment of, or early after infection in cynomolgus macaques is likely to facilitate a more favorable disease outcome.

Interestingly, some of the differential innate immune responses between the two species were observed before exposure to *Mtb*. This suggests that underlying factors inherent to the species, such as differences in genetics, may be responsible for the observed differences in immune responses (49). Alternatively, as cynomolgus and rhesus are typically bred separately, disparate exposure to environmental factors also may have shaped the diverging immune response to *Mtb* (50). A well-known manifestation of this phenomenon is trained immunity, which was not specifically addressed in this study but would be of interest for further research (26, 51). In that regard, the difference in pre-infection IgM response observed between the two species might be a result of differential exposure to non-tuberculous mycobacteria (NTM), even though all animals were screened negative for NTM-specific cellular immune responses prior to the start of the study. Lastly, another underlying factor influencing *Mtb* infection and disease susceptibility might be a dissimilar gut or pulmonary microbiome composition between the species (52–54). Further research will be required to unravel the possible mechanisms that connect nature and/or nurture with the early innate and pro-inflammatory immune response profiles in the context of the reduced susceptibility to TB disease displayed by cynomolgus macaques.

In conclusion, our study corroborates the differential phenotype between rhesus and cynomolgus macaques observed after *Mtb* infection, and points to early innate rather than adaptive immunity as a potential cause for this difference. These results provide novel leads for correlates of risk of developing TB disease after infection as well as clues for further research to identify the underlying mechanisms that result in these disparate immune responses between the two macaque species.

## Materials and Methods

### Ethics, Animals, and Handling

All housing and animal care procedures took place at the Biomedical Primate Research Center (BPRC) in Rijswijk, the Netherlands, and were in compliance with European directive 2010/63/EU, as well as the “Standard for Humane Care and Use of Laboratory Animals by Foreign Institutions” provided by the Department of Health and Human Services of the US National Institutes of Health (NIH, identification number A5539-01). The BPRC is accredited by the American Association for Accreditation of Laboratory Animal Care (AAALAC). Before the start of the study ethical approval was obtained from the independent animal ethics committee (in Dutch: Dierexperimentencommissie, DEC) as well as BPRC's institutional animal welfare body (in Dutch: Instantie voor Dierwelzijn, IvD). The study protocol was registered under DEC accession no. 761subA.

Ten male, non-Mauritian cynomolgus macaques (*Macaca fascicularis*) and 10 male Indian-type rhesus macaques (*Macaca mulatta*) were selected from the in-house colonies. Selected animals were screened as being negative for prior exposure to mycobacteria by means of tuberculin skin testing with Old Tuberculin (Synbiotics Corporation, San Diego, CA) and an IFNγ ELISPOT against Purified Protein Derivative (PPD) from *Mycobacterium bovis, Mycobacterium avium* (both Fisher Scientific, USA), or *Mycobacterium tuberculosis* (Statens Serum Institute, Copenhagen, Denmark). Selected cynomolgus and rhesus macaques were matched in age (mean age ± SD in years; 6.3 ± 0.69 vs. 6.1 ± 0.61, respectively) but differed significantly in weight (mean weight ± SD in kilograms; 7.6 ± 1.3 vs. 10.7 ± 2.8, *p* = 0.0029).

Throughout the experiment animals were socially housed (pair-wise) at biosafety level 3 and provided with enrichment in the form of food and non-food items on a daily basis. Diet was identical for both species. Animal welfare was monitored daily. Animal weight was recorded prior to each blood collection event. To limit possible discomfort due to severe TB disease humane endpoints were predefined prior to the start of the study. All animal handling and biosampling was performed under ketamine sedation (10 mg/kg, by intra-muscular injection). For endobronchial instillation ketamine (5 mg/kg) was supplemented with intramuscular medetomidine (0.04 mg/kg) and an analgesic sprayed into the larynx. By the end of the infection phase or when reaching a humane endpoint, animals were euthanized by intravenous injection of pentobarbital (200 mg/kg) under ketamine sedation.

Standard hematology on EDTA blood was performed on a Sysmex 2000i system (Siemens). CRP serum levels were determined using a Cobas™ Integra400+ (Roche Diagnostics). All veterinary staff and clinical lab personnel were blinded to animal treatment.

### *Mycobacterium tuberculosis* Infection and Monitoring of Infection Take

Animals were challenged with increasing doses of *Mycobacterium tuberculosis* Erdman K01 strain (BEI Resource, VA, USA). Each *Mtb* challenge dose was delivered by endobronchial instillation of 3 mL inoculum, targeting the lower left lung lobe and all challenge events occurred in a single session within 2–3 h from preparing the inoculum from a frozen *Mtb* stock, challenging all animals in random order. When applicable, all peripheral blood sampling and/or bronchoalveolar lavages were performed prior to administration of *Mtb*. For quality assurance and determination of final dose administered, serial dilutions from the inoculum preparation process of each challenge were plated on 7H10 Middlebrook plus PANTA antibiotic mixture plates (Tritium, the Netherlands). Doses reported in the Results section are the extrapolated doses calculated from the number of CFUs grown from the dilution closest to the inoculum (extrapolated doses administered: 0.2, 1.3, and 7 CFU).

Infection take was monitored by means of a non-human primate specific IFNγ ELISPOT (U-CyTech, the Netherlands) performed 3 and 4 weeks each after *Mtb* exposure. Assay was performed according to manufacturer's protocol. In brief, 200,000 PBMCs were incubated in triplicate for 24 h with *Mtb*-derived PPD (Statens Serum Institute, Denmark) or recombinant ESAT6-CFP10 fusion protein (provided by K. Franken from the Ottenhoff lab, Leiden University Medical Center) (34, 55), both at a final concentration of 5 μg/mL. The following day cells were washed and transferred to anti-IFNγ coated membrane plates (Millipore). After 24 h of incubation cells were discarded and membrane-bound IFNγ was visualized with biotinylated anti-IFNγ detector antibody, streptavidin-horseradish peroxidase conjugate and tetramethylbenzidine substrate. Spots were quantified using an automated reader (AELVIS, Hannover). The pre-infection response to both PPD and ESAT6-CFP10 of all animals was used to determine the threshold for conversion from negative to positive. Cut-off was set at the average medium control corrected pre-infection response plus 3x the standard deviation. This resulted in a cut-off value for positivity of 50 control corrected spots per million for PPD as well as ESAT6-CFP10. Animals were considered infected if either the PPD or ESAT6-CFP10 response reached the threshold for positivity.

### Peripheral and Local Bio-Sample Collection and Processing

Cells from the pulmonary mucosa were harvested by broncho-alveolar lavage (BAL), targeting the lower left lung lobe. Three volumes of 20 mL of prewarmed 0.9% saline solution were consecutively instilled and recovered. BALs were passed over a 100 μm filter and cellular fraction was separated from fluid by centrifugation for 10 min at 400 g. Supernatant was decanted and stored at −80°C pending further analysis. Cell pellet was resuspended in RPMI supplemented with 10% fetal calf serum (FCS), glutamax and penicillin/streptomycin (from now on referred to as R10). Heparinized blood for immune monitoring, EDTA blood for standard hematology, and serum for clinical chemistry were all collected by venipuncture. PBMCs were obtained by density gradient centrifugation of heparinized blood. Because of a species difference in blood osmolality we used Lymphoprep lymphocyte separation medium (Axis-Shield, UK) for rhesus macaques and a Percoll solution (GE Healthcare, IL, USA) for cynomolgus macaques. After harvesting and washing cells were resuspended in R10 for downstream immunological assays. Serum tubes were centrifuged for 10 min at 1,000 g to harvest cell-free serum which was stored at −80°C pending further analysis. Serum and BAL fluid were filter-sterilized by centrifugation through 0.2 μm PVDF membrane plates (Fisher Scientific) before analysis.

### Post-mortem Tuberculosis Pathology Assessment

After euthanasia tuberculosis pathology was assessed by a semi-quantitative grading system [adapted from (56)] based on lesion size, manifestation and frequency, and lymph node involvement. The thoracic cavity, including the heart, ribcage, vertebrae, and diaphragm were all macroscopically scored for the presence of granulomas and pleural adhesions. Lungs were isolated and lobes were separated from the trachea. Subsequently, lung lobes were cut in 5 mm thick slices and scored for the amount of pathology. The most affected lung lobe was designated as the primary lung lobe. From this lobe individual lesions were isolated, weighed, and processed separately. The remainder of the primary lobe was mechanically homogenized. The remaining lung lobes were pooled and mechanically homogenized. Lung draining lymph nodes were isolated from the trachea and scored for size and extent of involvement. Axillar and inguinal lymph nodes were similarly assessed. Extra-thoracic organs such as the kidneys, spleen, pancreas, and liver were macroscopically assessed for the presence of lesions. The summed score of all extra-thoracic organs was used as a measure of extra-thoracic dissemination.

### Determination of Bacterial Load in Tissue

After pathology assessment and initial homogenization lesions and lung lobes were further homogenized in GentleMACS C- and M-tubes (Miltenyi). Bronchoalveolar lymph nodes were first processed to a single cell suspension by straining over a 100 μm cell strainer (Greiner) and subsequently homogenized in GentleMACS M-tubes. All homogenates were frozen at −80°C before quantification of bacterial counts. Thawed samples were 3-fold serially diluted and plated on 7H10 plates supplemented with PANTA antibiotic mixture (Tritium, the Netherlands). Plates were incubated at 37°C for at least 3 weeks until CFU were observed. Plates were incubated for at least 6 weeks before scored as negative. CFUs were counted with a Scan4000 automated counting system (Interscience, France).

### Mycobacterial Growth Inhibition Assay

Cryopreserved PBMCs were thawed and incubated with 10 U/mL benzonase (Merck) in R10. After 2 h cells were washed, taken up in RPMI supplemented with 10% inactivated pooled human serum and glutamax and counted. 1 × 10^6^ PBMCs were co-cultured with 100 CFU BCG Pasteur (P3) for 4 days, in a final volume of 600 μl, on a rotator at 37°C, as described in Joosten et al. (26).

After 4 days, samples were transferred to Mycobacterial Growth Indicator Tubes (MGIT, Beckton Dickinson) supplemented with PANTA antibiotic mixture and OADC enrichment. Tubes were placed in a BACTEC960 system (Beckton Dickinson) and measured until Time to Positivity (TTP) was reached. Samples with a TTP <100 h were considered to be contaminated and excluded from analysis.

To be able to convert TTP to CFU, serial dilutions of the BCG stock were plated on Middlebrook 7H10 plates to determine CFU, as well as added to MGIT tubes to determine TTP. Counted CFUs were converted to 10log CFU and a standard curve to convert TTP to logCFU was generated by means of linear regression.

### Flow Cytometric Analysis of Local and Peripheral Immune Subsets

Whole blood, PBMCs and BAL cells were profiled by flow cytometry to characterize monocyte phenotype and cytokine production by T-cells and APC (see Supplementary Table 1 for overview of panels used).

#### T-Cell Cytokine Production

Cryopreserved BAL cells and PBMCs were thawed in R10 containing 50 U/mL benzonase. After washing cells were incubated with either medium or 5 μg/mL PPD for 3 h at 37°C. GolgiPlug transport inhibitor (BD Biosciences) was added and incubated overnight. Cells were stained the subsequent day with the T-cell cytokine panel (Supplementary Table 1). PMA/ionomycin stimulated samples were taken along as technical/positive controls.

#### APC Cytokine Production

Two hundred microliter heparinized whole blood was incubated with medium or 25 ug/mL *Mtb* H828 WCL (BEI Resource, VA, USA) 37°C for 3 h and subsequently incubated overnight with Golgiplug transport inhibitor. Prior to staining with the APC cytokine panel (Supplementary Table 1) samples were treated with Pharm Lyse (BD Biosciences) to lyse red blood cells.

#### Pro- and Anti-inflammatory Monocyte Phenotyping

Two hundred microliter heparinized whole blood was stained *ex vivo* with the monocyte phenotyping panel (Supplementary Table 1). After staining cells were treated with FACS Lyse (BD Biosciences) to lyse red blood cells.

After staining all samples were fixed overnight with 2% paraformaldehyde and measured on a 3 laser, 14 color LSR-II flowcytometer (BD Biosciences). All analyses were performed with FlowJo software version 10 (Treestar). Any anomalies indicative of unstable signal acquisition were excluded using the time parameter. Representative gating strategies are depicted in Supplemental Figure 3.

### Antibody ELISAs

*Mtb*-reactive antibody levels in serum and BAL were determined by Enzyme Linked ImmunoSorbent Assay (ELISA). In brief, 96-well plates were coated with 5 μg/mL *Mtb* HN828 WCL (BEI Resource, VA, USA) in PBS. After overnight blocking with 1% BSA, serial dilutions of BAL and serum samples were added and incubated for 2 h at 37°C. Bound antibodies were subsequently detected with horse radish peroxidase-conjugated anti-IgG (Rockland, PA, USA) in combination with tetramethylbenzidine substrate, alkaline phosphatase-conjugated anti-IgA (Fisher Scientific), or alkaline phosphatase-conjugated IgM (Sigma) with *para*-nitrophenylphosphate substrate for ELISA color development. All samples were normalized to arbitrary units (AU) against a serial dilution of a positive reference sample included in all assays.

### Multiplex Cytokine Assay

Cytokine production of BAL cells stimulated overnight with PPD (5 μg/mL) and *Mtb* WCL (25 μg/mL) was assessed by a custom Procartaplex Luminex kit (ThermoFisher Scientific, USA). Assays were performed according to manufacturer's protocol. In short: supernatants of stimulated cells were incubated with beads coated with cytokine-specific antibodies. Bound cytokines were visualized using biotin-coupled detection antibodies and PE-labeled streptavidin. Beads were acquired on a Bioplex 200 system and cytokine levels were calculated with Bioplex Manager software version 6.1 (both Biorad, CA, USA).

### Statistics

Statistical analyses were performed using Graphpad Prism software version 7. Significance of differences between groups was calculated by two-sided Mann-Whitney testing. Paired observations within groups were analyzed by two-sided Wilcoxon signed-rank test. Unless indicated otherwise in the figure legends, statistical calculations are based on *n* = 10 observations per treatment group.

## Data Availability Statement

The data sets generated and analyzed in this present study are available from the corresponding authors upon reasonable request. Likewise, upon request biomaterials that are still available from this study could be shared for further research.

## Ethics Statement

The animal study was reviewed and approved by the Dutch independent animal ethics committee (in Dutch: Dierexperimentencommissie, DEC) as well as BPRC's institutional animal welfare body (in Dutch: Instantie voor Dierwelzijn, IvD).

## Author Contributions

Study conceptualization by FV. Study design and experimental methodology conceived by KD and FV. KD, RV, CS, CB, SH, KM, KH, and MV were involved sample collection and processing and downstream immunological and bacteriological analyses. RV and KH were responsible for general project management. CK bore overall responsibility as chair of the BPRC's Department of Parasitology. KD and FV wrote the manuscript. TO advised on immune analyses and reviewed and edited the manuscript.

### Conflict of Interest

The authors declare that the research was conducted in the absence of any commercial or financial relationships that could be construed as a potential conflict of interest.
