# Supplementary material for: Disparate Tuberculosis Disease Development in Macaque Species Is Associated With Innate Immunity
Source: Front Immunol. 2019 Nov 1;10:2479. doi: 10.3389/fimmu.2019.02479 (PMC6838139; doi:10.3389/fimmu.2019.02479)
Supplement: Supplementary file 1 [file Data_Sheet_1.docx]

**Supplementary Data**

**Supplementary Figure 1: Additional parameters of tuberculosis disease severity**


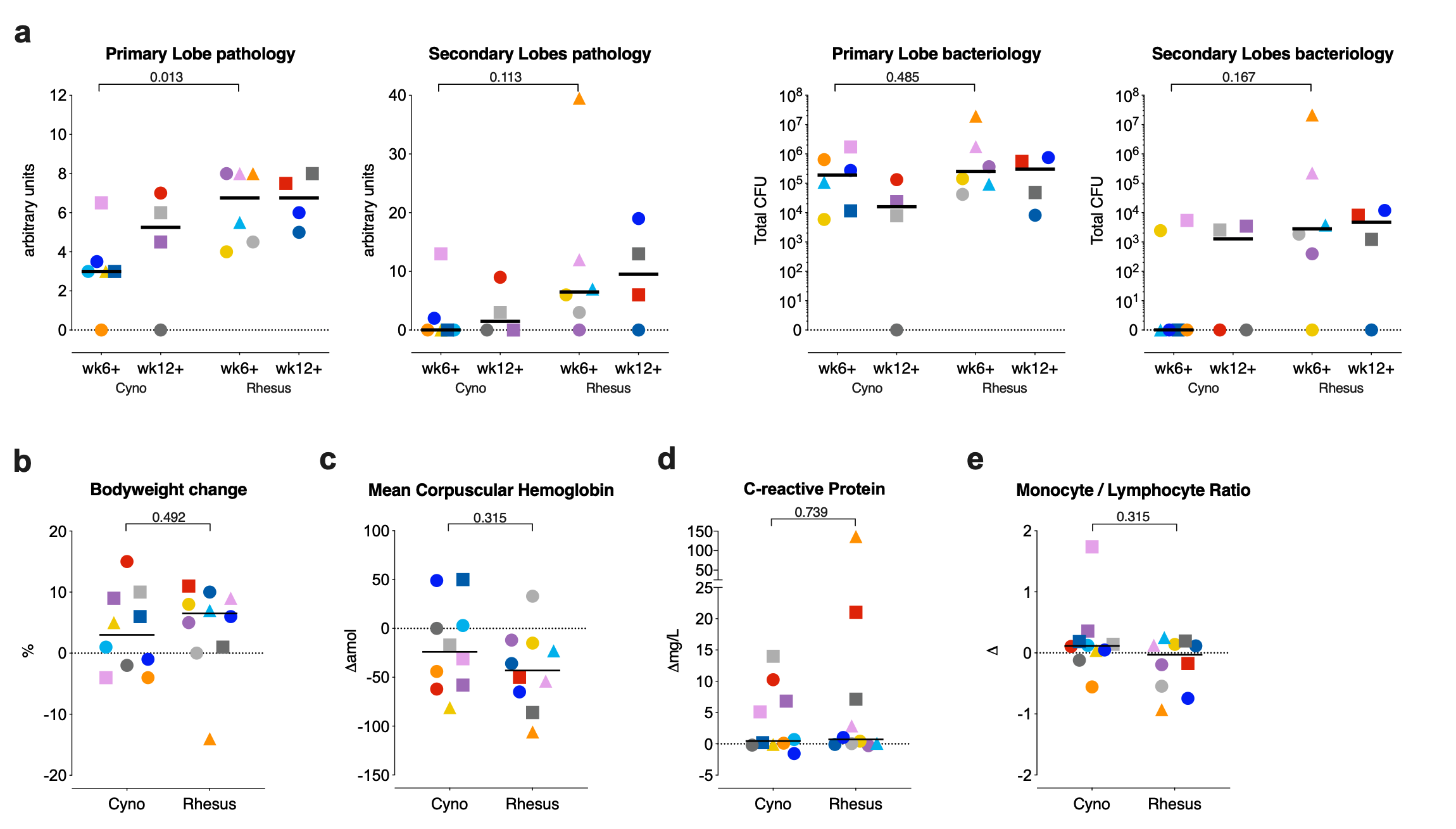


(**a**) Lung pathology plotted per species per time-to-endpoint. (**b-e**) Change in (**b**) bodyweight and (**c-e**) haematological and serological parameters associated with tuberculosis disease over the course of the *Mtb* infection. Circles represent animals infected after administration of 1.3 CFU, squares represent animals infected after administration of 7 CFU and triangles indicate animals administered both 1.3 and 7 CFU of *Mtb.* N=10 animals per group. Horizontal lines indicate group medians. Two-sided Mann-Whitney testing was used to determine significance of differences between groups. Colour coding per individual, as defined in Table 1, is consistently applied throughout

**Supplementary Figure 2: Peripheral and local CD4 cytokine production**

**
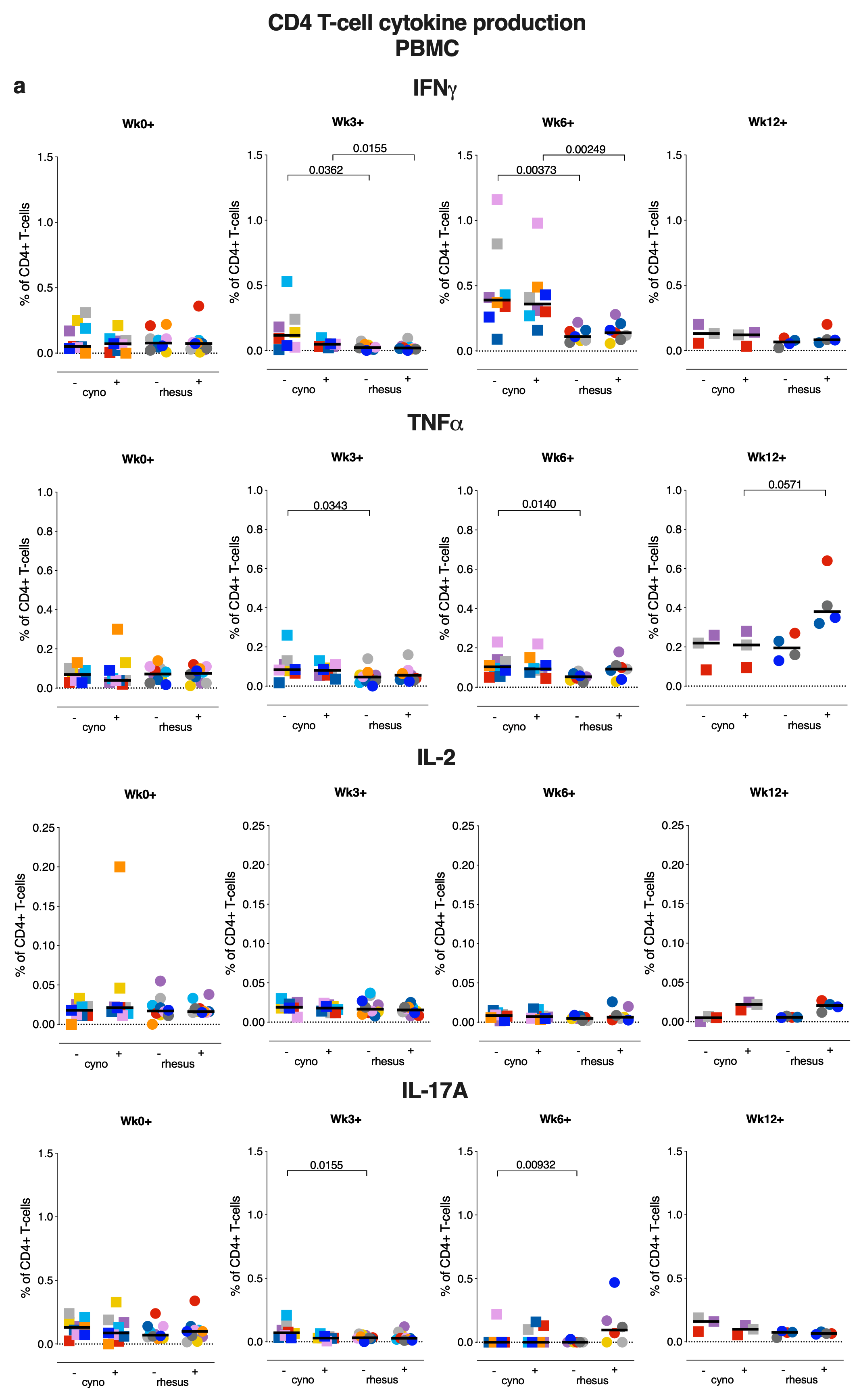
**

**Supplementary Figure 2:** (Continued)


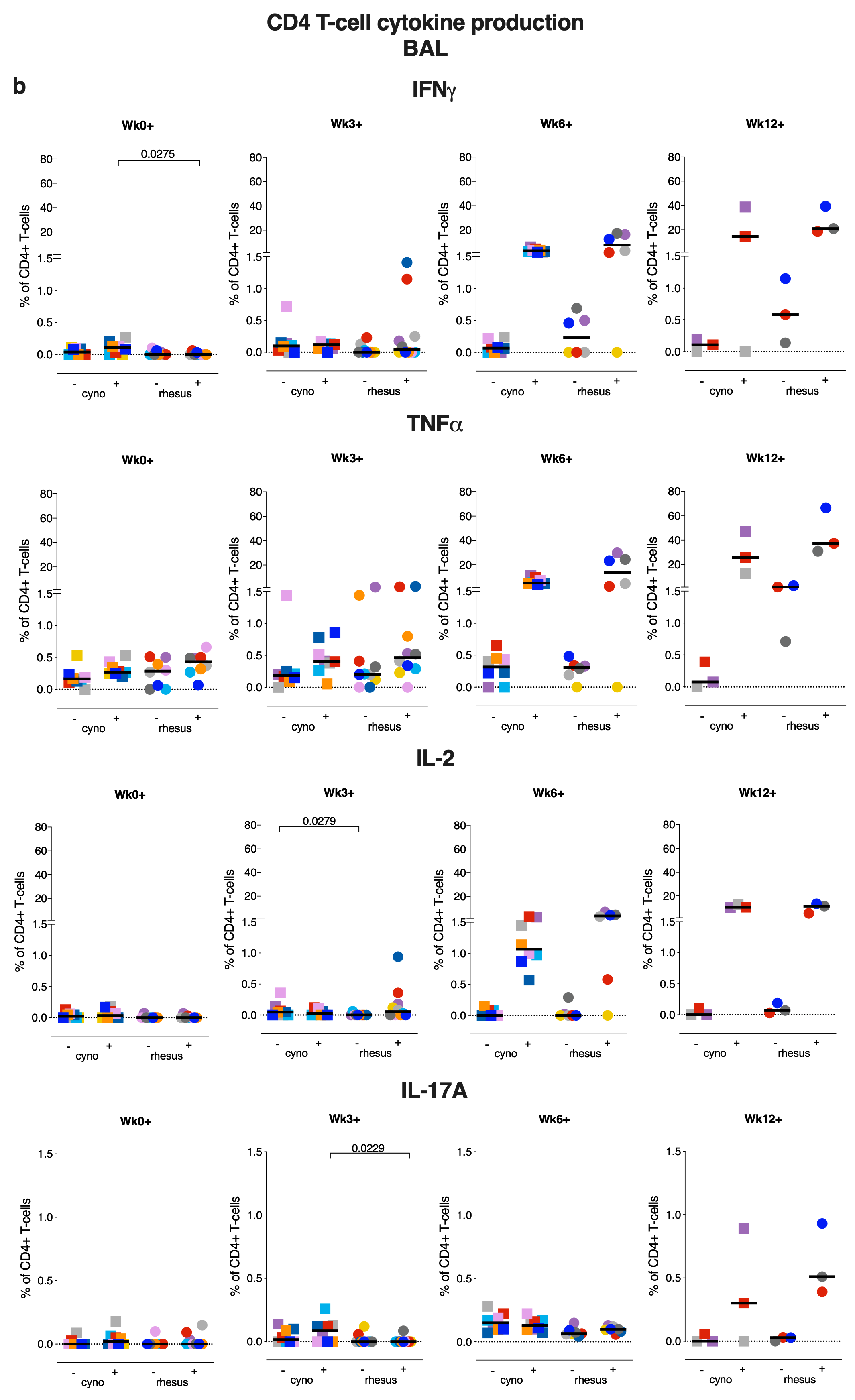


**Supplementary Figure 2:** (**a**) percentage CD4 T-cells in the periphery producing IFNγ, TNFα, IL2 or IL17A after overnight incubation with culture medium (-) or PPD (+)

(**b**) percentage CD4 T-cells at the pulmonary mucosa producing IFNγ TNFα, IL2 or IL17A after overnight incubation with culture medium (-) or PPD (+)

Data are aligned to the moment of each individual's time point of infection take. Number of animals per time point varies due to sample availability. Circles represent animals infected after administration of 1.3 CFU, squares represent animals infected after administration of 7 CFU and triangles indicate animals administered both 1.3 and 7 CFU of *Mtb.* Horizontal lines indicate group medians. P-values of possible differences between species were determined by two-sided Mann-Whitney testing.

**Supplementary Figure 3: Gating strategy applied for flowcytometric analysis of monocytes and T-cells**

**A**

**
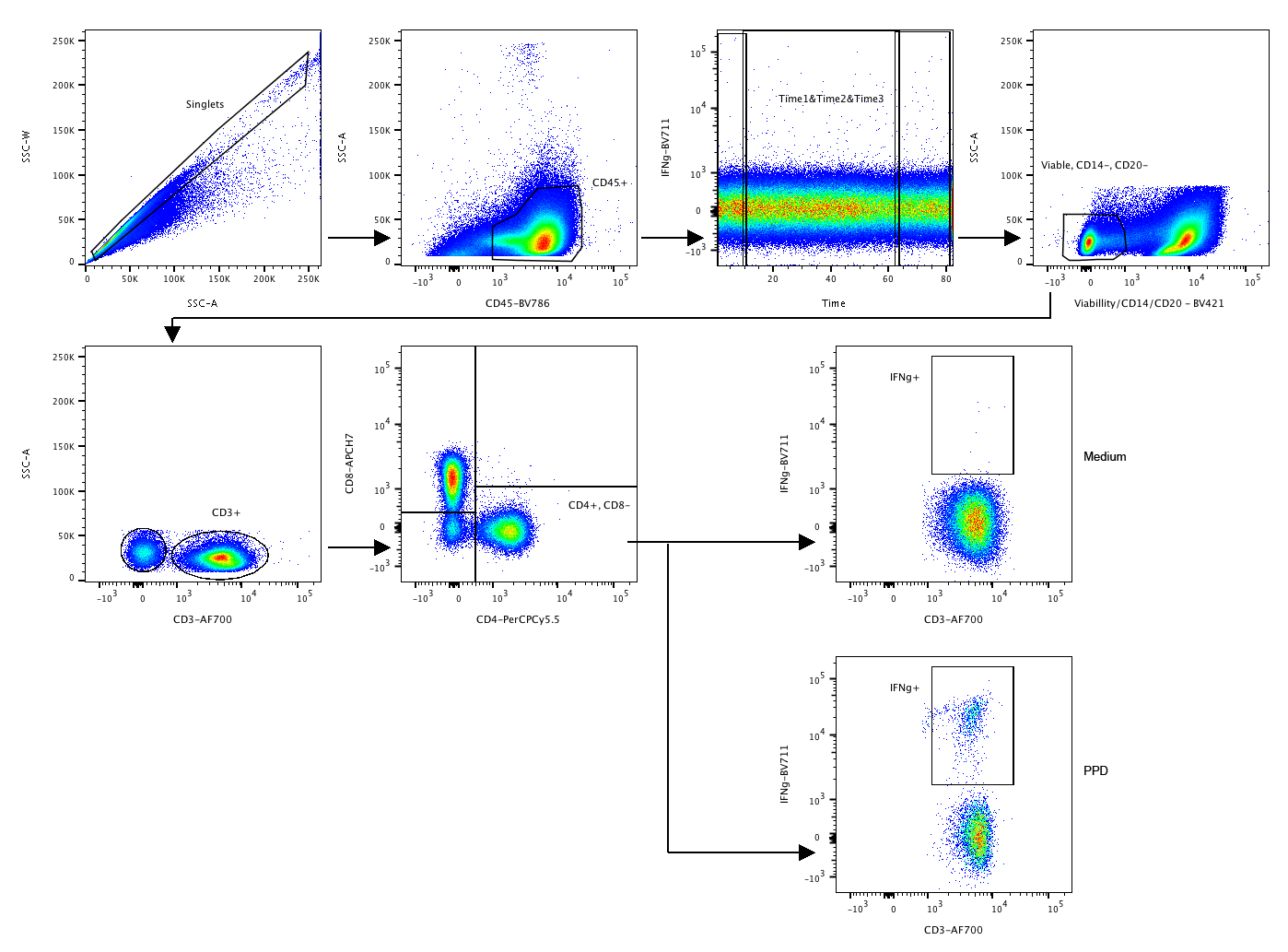
**

**B**

**
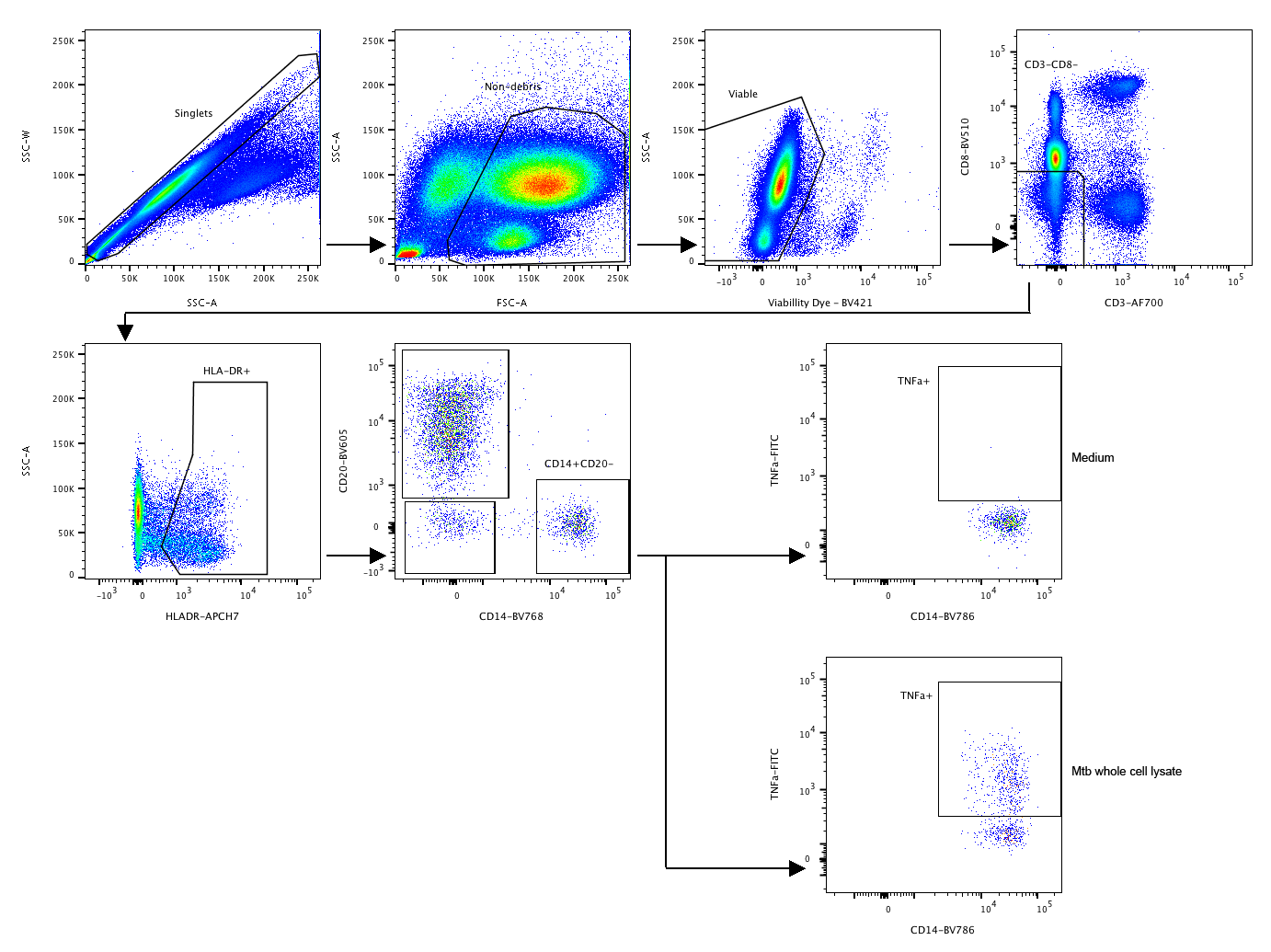
**

**Supplementary Figure 3:** (Continued)

**C**

**
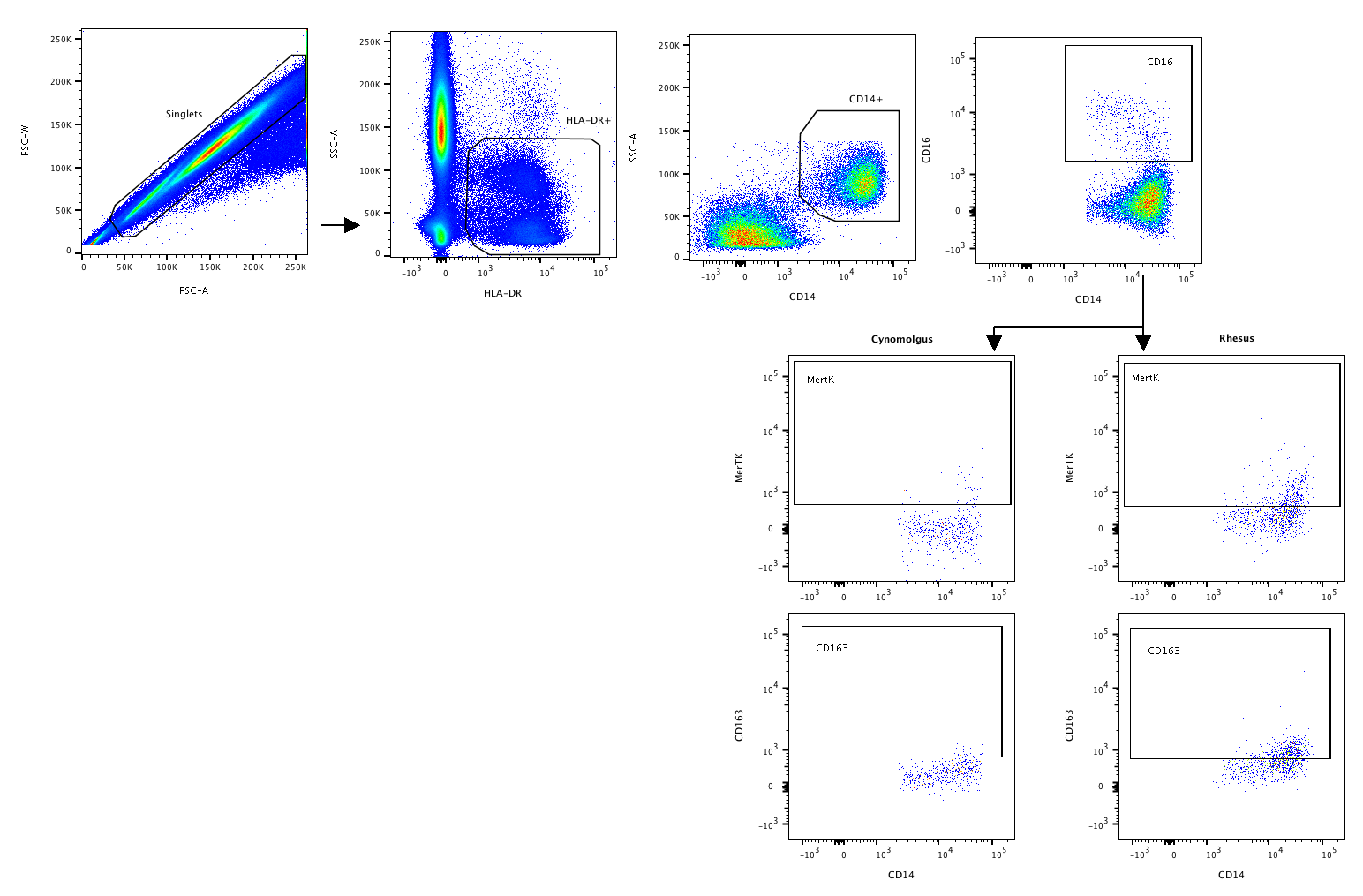
**

**Supplementary Figure 3:** Representative gating strategies of the analysis of **a**) T-cell cytokine production by medium and PPD-stimulated PBMCs (depicted here) and BAL cells, **b**) cytokine production by monocytes after medium or Mtb Whole Cell Lysate stimulation of whole blood and **c**) *ex vivo* MerTK and CD163 expression of monocytes.

**Supplementary Table 1:**
